# Deceptive Desmas: Molecular Phylogenetics Suggests a New Classification and Uncovers Convergent Evolution of Lithistid Demosponges

**DOI:** 10.1371/journal.pone.0116038

**Published:** 2015-01-07

**Authors:** Astrid Schuster, Dirk Erpenbeck, Andrzej Pisera, John Hooper, Monika Bryce, Jane Fromont, Gert Wörheide

**Affiliations:** 1 Department of Earth- & Environmental Sciences, Palaeontology and Geobiology, Ludwig-Maximilians-Universität München, Richard-Wagner Str. 10, 80333 Munich, Germany; 2 SNSB – Bavarian State Collections of Palaeontology and Geology, Richard-Wagner Str. 10, 80333 Munich, Germany; 3 GeoBio-Center^LMU^, Ludwig-Maximilians-Universität München, Richard-Wagner Str. 10, 80333 Munich, Germany; 4 Institute of Paleobiology, Polish Academy of Sciences, ul. Twarda 51/55, 00-818 Warszawa, Poland; 5 Queensland Museum, PO Box 3300, South Brisbane, QLD 4101, Australia; 6 Eskitis Institute for Drug Discovery, Griffith University, Nathan, QLD 4111, Australia; 7 Department of Aquatic Zoology, Western Australian Museum, Locked Bag 49, Welshpool DC, Western Australia, 6986, Australia; University of Texas, United States of America

## Abstract

Reconciling the fossil record with molecular phylogenies to enhance the understanding of animal evolution is a challenging task, especially for taxa with a mostly poor fossil record, such as sponges (Porifera). ‘Lithistida’, a polyphyletic group of recent and fossil sponges, are an exception as they provide the richest fossil record among demosponges. Lithistids, currently encompassing 13 families, 41 genera and >300 recent species, are defined by the common possession of peculiar siliceous spicules (desmas) that characteristically form rigid articulated skeletons. Their phylogenetic relationships are to a large extent unresolved and there has been no (taxonomically) comprehensive analysis to formally reallocate lithistid taxa to their closest relatives. This study, based on the most comprehensive molecular and morphological investigation of ‘lithistid’ demosponges to date, corroborates some previous weakly-supported hypotheses, and provides novel insights into the evolutionary relationships of the previous ‘order Lithistida’. Based on molecular data (partial mtDNA CO1 and 28S rDNA sequences), we show that 8 out of 13 ‘Lithistida’ families belong to the order Astrophorida, whereas Scleritodermidae and Siphonidiidae form a separate monophyletic clade within Tetractinellida. Most lithistid astrophorids are dispersed between different clades of the Astrophorida and we propose to formally reallocate them, respectively. Corallistidae, Theonellidae and Phymatellidae are monophyletic, whereas the families Pleromidae and Scleritodermidae are polyphyletic. Family Desmanthidae is polyphyletic and groups within Halichondriidae – we formally propose a reallocation. The sister group relationship of the family Vetulinidae to Spongillida is confirmed and we propose here for the first time to include *Vetulina* into a new Order Sphaerocladina. Megascleres and microscleres possibly evolved and/or were lost several times independently in different ‘lithistid’ taxa, and microscleres might at least be four times more likely lost than megascleres. Desma spicules occasionally may have undergone secondary losses too. Our study provides a framework for further detailed investigations of this important demosponge group.

## Introduction

### Background

Demospongiae Sollas, 1885 [1], with more than 85% of all living species, represents the largest and morphologically most diverse group of the phylum Porifera [2]. Today demosponges encompass 15 orders, and more than 8,500 accepted extant species [3]. Recent molecular evidence was pivotal in the classification of Demospongiae into four major clades: Keratosa Grant, 1861 [4], Verongimorpha Erpenbeck et al., 2012 [5], Haploscleromorpha Cárdenas et al., 2012 [6] and Heteroscleromorpha Cárdenas et al., 2012 [6], [7] – the latter representing the largest and evolutionary most important group within demosponges [6]. ‘Lithistida’ Schmidt, 1870 [8], on the other hand, until now remained a highly problematic and likely polyphyletic group of living and fossil sponges, and indeed provides the richest fossil records of all Porifera. Lithistid sponges differ from other demosponges by the unique possession of choanosomal spicules called desmas. These have been defined as “articulating choanosomal megascleres of various geometry and usually complex morphology, often secondarily modified and very irregular” [9]. Most living and fossil desma-bearing demosponges have a solid, rigid, heavily silicified skeleton – an important feature used in the morphological-based classification [9] – but a much fewer number of species have sparse, disarticulated desmas scattered throughout the mesohyl of their otherwise compressible choanosomal skeleton [10], [11].

Compared to the lithistid fossil record (34 families, >300 genera [12]), the diversity of Recent species is comparatively poor (13 families and 41 genera, including five poorly known and of uncertain status) [9]. However, the Recent diversity of lithistids might extend back to the late Mesozoic in Europe (AP, unpublished results), suggesting that Recent lithistids are severely understudied [13]. ‘Lithistida’ inhabit tropical, subtropical and temperate regions from shallow waters to the deep sea, where they usually form faunal assemblages with other demosponges and, in the deep sea, also with hexactinellid sponges. Frequently, lithistid sponges occur on marine seamounts, their vertical slopes, on margins of continental shelves [14], and are common in submarine caves, e.g. in the Mediterranean [15], [16] and shallow lava tubes in French Polynesia. [17]. Furthermore, some lithistids such as e.g. *Theonella swinhoei*, *Discodermia polydiscus, Discodermia dissoluta,* produce a wide range of bioactive compounds [18], [19] and therefore are of special interest to the biomedical industry.

### Historic taxonomic overview on lithistid demosponges

**Figure 1:**
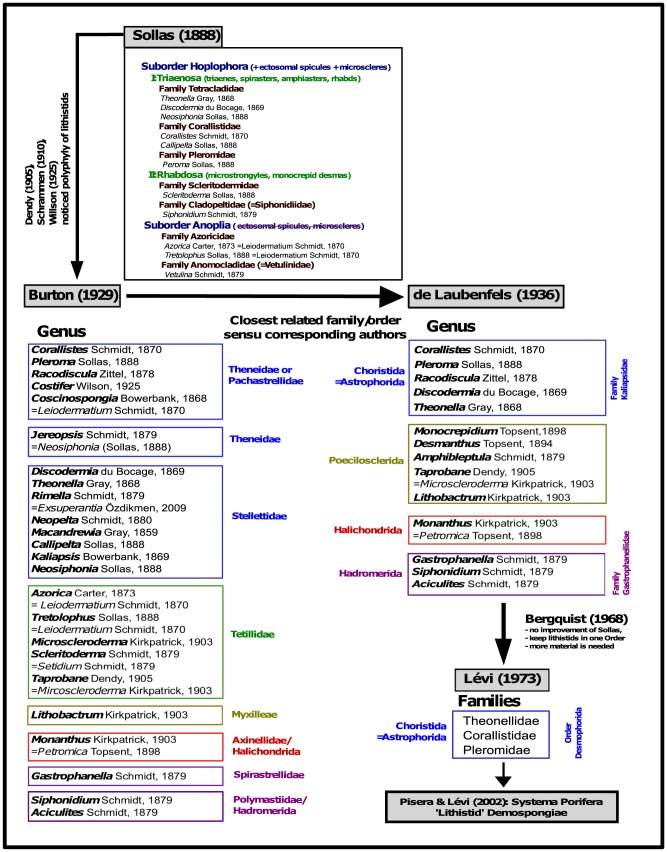
Historic taxonomic overview of lithistid demosponges. From the monophyly suggested by Sollas (1888) to the hypotheses of polyphyly of modern authors, it shows the attempts to reallocate most genera of the order ‘Lithistida’ to their closest relatives.

Sollas (1888) [20] undertook the first comprehensive taxonomic study of lithistid sponges, based mainly on the presence or absence of ectosomal spicules and microscleres. He created two suborders Hoplophora and Anoplia (see Fig. 1), and considered that desmas occurred as a single evolutionary event. Lithistids were suggested to form a monophyletic group together with the Choristida ( = Astrophorida), with the Anoplia considered to be the end lineage with the loss of all ectosomal spicules and microscleres. Dendy (1905) [21], Schrammen (1910) [22] and Wilson (1925) [23], however, suggested ‘Lithistida’ were polyphyletic and criticized Sollas’ classification for excluding microscleres within the concept of his suborder Anoplia. Burton (1929) [24] was the first who attempted to reallocate many lithistid genera to their closest non-lithistid families based on alleged morphological characters of the Theneidae, Pachastrellidae, Stellettidae (Choristida,  =  now Astrophorida), Tetillidae (Spirophorida), “Myxilleae” (Poecilosclerida), Axinellidae (Halichondrida), Spirastrellidae and Polymastiidae (Hadromerida) *sensu* Burton (1929). This classification was refined by de Laubenfels (1936) [25], who established two new families: Kaliapsidae for the genera belonging to Choristida and Gastrophanellidae for genera showing affinities to the order Hadromerida. Although both classifications show conflicting results within some genera (e.g. in *Microscleroderma*), several hypotheses were similar regarding the reallocation of many lithistid taxa (see Fig. 1). Bergquist (1978) [26] subsequently argued that Burton’s and de Laubenfels’ hypotheses were based on weak assumptions and more material and detailed descriptions would be needed to unequivocally allocate those lithistid sponges to their closest relatives. Lévi (1973) [27] followed Burton’s and de Laubenfels’ assumptions and stated that all lithistid genera belonging to the families Theonellidae, Corallistidae and Pleromidae should be placed within the Choristida under the name Desmophorida. Nevertheless, he also emphasized the uncertainty of relationships between the remaining ‘non-choristid’ lithistids [27], [28].

Despite their long acknowledged polyphyly, and all these attempts to reallocate lithistids to alleged sister-taxa in the past, lithistid Demospongiae were maintained in a single ‘order’ of demosponges within the most recent comprehensive taxonomic revision of Porifera, the *Systema Porifera* [9]. This was primarily due to the many still-unresolved or contested phylogenetic hypotheses throughout the families of ‘Lithistida’ and incomplete independent (e.g. molecular) evidence to support or refute particular hypotheses across the ‘order’.

The current classification *sensu Systema Porifera* [9] comprises 13 families: Azoricidae Sollas, 1888 [20], Corallistidae Sollas, 1888 [20], Desmanthidae Topsent, 1894 [29], Isoraphiniidae Schrammen, 1924 [30], Macandrewiidae Schrammen, 1924 [30], Neopeltidae Sollas, 1888 [20], Phymaraphiniidae Schrammen, 1910 [22], Phymatellidae Schrammen, 1910 [22], Pleromidae Sollas, 1888 [20], Scleritodermidae Sollas, 1888 [20], Siphonidiidae Lendenfeld, 1903 [31], Theonellidae Lendenfeld, 1903 [31] and Vetulinidae Lendenfeld, 1903 [31]. ‘Lithistida’ has been shown to be polyphyletic based on morphology [22], [25], [28], [32], [33], [34] and limited molecular datasets [35], [36], [37], [38], [39], but until now not in an integrative dataset including both morphology and molecular characters.

### Morphological spicule arrangements of lithistids and spicule evolution within demosponges

**Figure 2:**
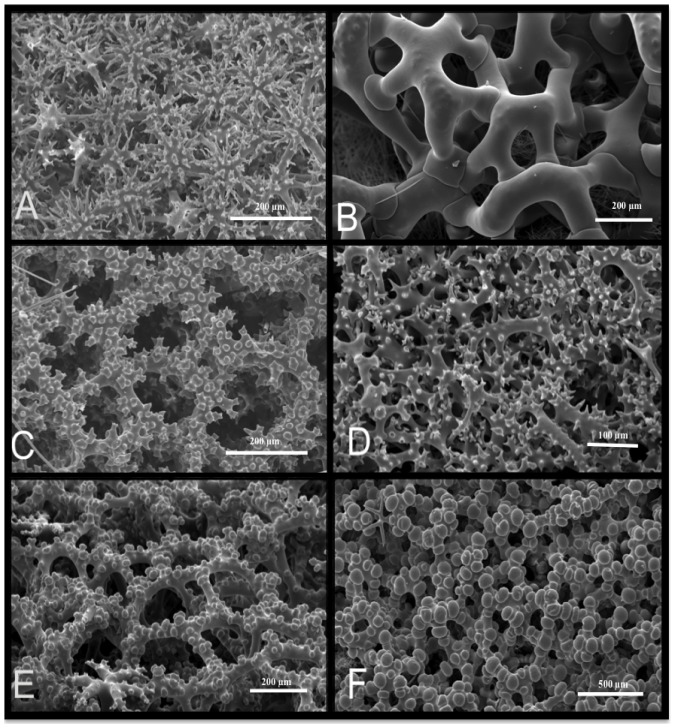
Various desma skeletons within lithistid demosponges. (A) sphaeroclone desmas (Vetulinidae); (B) megaclone desmas (Pleromidae); C–D rhizoclone desmas (Scleritodermidae, Azoricidae, Siphonididae); E–F dicranoclone desmas (Corallistidae).

**Figure 3:**
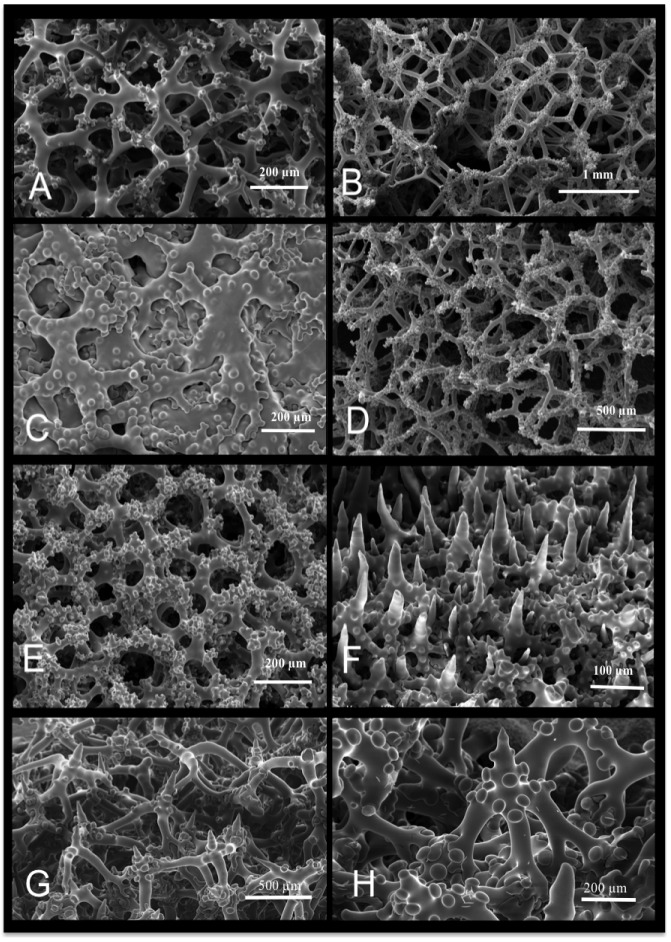
Various desma skeletons within lithistid demosponges. (A) tetraclone desmas (Phymatellidae); (B) tetraclone desmas (Theonellidae); (C–D) monaxial complex shaped desmas (Neopeltidae); (E) complex shaped desmas (Macandrewiidae) resembling tetraclones; (F) trider-like desmas of Desmanthidae; (G–H) trider-like desmas of Phymaraphiniidae.

**Figure 4:**
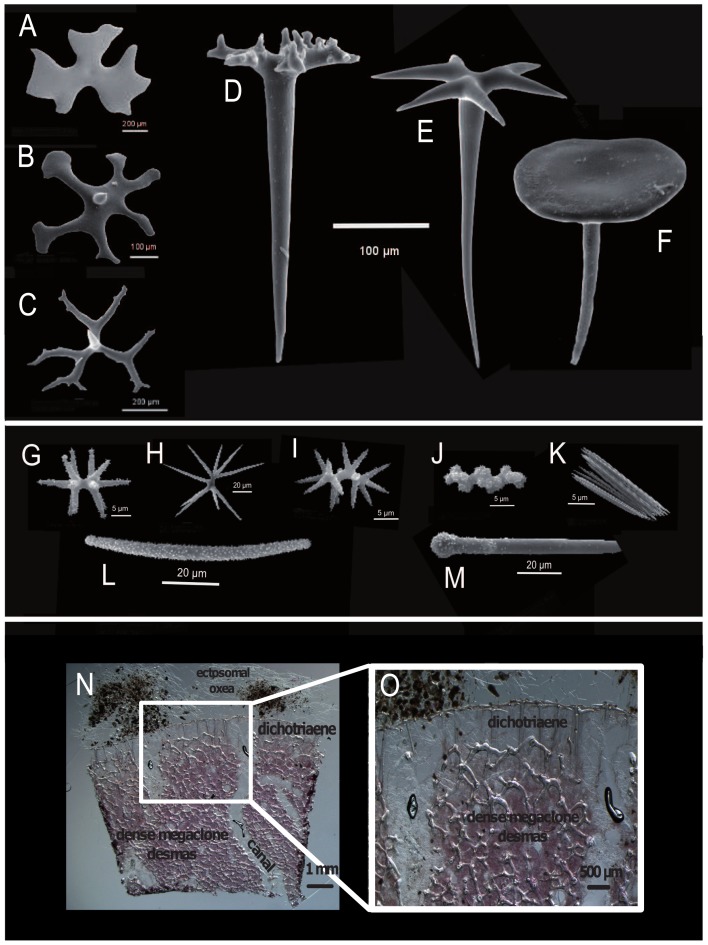
Illustration of different mega- and microscleres within lithistid demosponges. (A–F) different types of ectosomal spicules. (A): Monaxial ectosomal plate as found in the family of Neopeltidae. (B,C): Different phyllotriaenes within the family Theonellidae. (D,E): Two representatives of dichotriaenes (D): *Neophrissospongia*, (E): Corallistidae. (F): Discotriaene as found in the family Theonellidae. **(G–M) different types of microscleres.** (G): Amphiaster (Neopeltidae). (H): Metaster (Corallistidae). (I,J): Spiraster (Corallistidae). (K): Raphids (Azoricidae). (L): acanthorhabds (Scleritodermidae). (M): Exotylostyl (Siphonididae). (N,O) cross-sections of the ectosome and upper part of choanosome showing the skeleton architecture within the family Pleromidae. (N) *Pleroma turbinatum* collected during the Deep Down Under Expedition in 2009 at the deep fore-reef slopes of the Osprey Reef (Coral Sea, Australia).

Lithistid sponges present a wide array of monaxial (Fig. 2 B; Fig. 2 C, D, F), tetraxial (Fig. 2 C–F; Fig. 3 A,B,E) and polyaxial (Fig. 2 A) desma spicules as well as desma spicules which can be disarticulated (Fig. 4 D). Ectosomal megascleres may consist of phyllo-, disco-, dicho- and anatriaenes, rhabds, and oxeas (Fig. 4), and microscleres may include amphiasters, spirasters, microxeas, raphides (Fig. 4) and/or sigmaspires. A typical lithistid skeletal architecture of ectosomal megascleres is illustrated by *Pleroma turbinatum* (Fig. 4 N,O), with oxeas protruding from the choanosome followed by a layer of dichotriaenes in the ectosomal skeleton and dense megaclone desmas within the choanosomal skeleton.

Lithistid demosponges also present a high diversity of desma morphologies, megascleres, microscleres and skeletal structures. For example, *Neoschrammeniella norfolki* Schlacher-Hoenlinger, Pisera & Hooper, 2005 (Family Corallistidae) can have up to six different spicule types including megascleres and microscleres. Hence, this broad spicule diversity within lithistids and other astrophorids can be used as an appropriate tracer to study spicule evolution within demosponges. The importance of spicule homoplasy (convergent evolution and secondary losses) within demosponges is well known from several studies based on morphological and molecular characters in a variety of different sponge taxa: such as *Crambe crambe* [40], [41], in the order Astrophorida [36], [42] and many other Heteroscleromorpha [7]. It is also well known that secondary character losses in phylogenetic studies can have a fundamental influence in understanding conflicting molecular and morphological datasets [43]. However, due to low spicule diversity and few morphological characters in most non-lithistid demosponges, except for those belonging to Tetractinellida (Astrophorida + Spirophorida) [44], only little is known on how frequent secondary losses have occurred throughout Demospongiae. Recent molecular and morphological analyses of the Astrophorida [36] emphasized the repeated occurrence of secondary losses of both spicule types, megacleres and microscleres, and concluded that this evolutionary process is more common in demosponges than previously thought.

### State of knowledge on the molecular phylogeny of lithistid sponges

The first molecular investigations focusing on lithistid sponges were based on a small fragment of the 18S rDNA gene (550 bp), comprising nine species representing seven different families [14]. However, the final outcome of this study was hampered due to the low variation within the selected gene region and the small taxa-set [38]. While there is a growing number of molecular phylogenies of Demospongiae using different molecular markers only few species of lithistids were included [7], [36], [38], [42], [45], [46], [47], [48]. The broadest molecular dataset for lithistid sponges was assembled during the Porifera Tree of Life project, based on a nearly complete small-subunit ribosomal 18S rDNA gene, and it included 29 specimens from 12 different genera and six families [39]. Table 1 summarizes the current molecular data available in NCBI GenBank (www.ncbi.nlm.nih.gov/genbank) for lithistid sponges (16 genera from 9 families), together with their suggested reallocation to their closest non-lithistid relatives. However, this sample size is still very small compared to the currently approx. 300 Recent described ‘valid’ species in the World Porifera Database [44], from 41 genera (plus five genera of uncertain status) and 13 families. In summary phylogenetic relationships of lithistid demosponges with non-lithistid species based on morphological data remains mostly speculative and untested by more substantial independent molecular evidence.

**Table 1:** The current molecular data for lithistid demosponges from GenBank, and their suggested reallocation of 9 of the 13 lithistid families to their closest non-lithistid relatives.

Lithistid taxa	Gene Region	Reallocation	References
**Azoricidae**			
* Leiodermatium*	18S	Tetractinellida	[14]*
**Corallistidae**			
* Corallistes*	18S, 28S, CO1, ITS	Astrophorida	[39], [45], [46]
* Neophrissospongia*	18S, 28S, CO1	Astrophorida	[36], [39]
**Desmanthidae**			
* Desmanthus*	28S, 18S	Dictyonellidae	[39], [49]
* Petromica*	18S	Halichondriidae	[14], [39]
**Neopeltidae**			
* Callipelta*	28S, 18S	Astrophorida	[39], [50]
* Homophymia*	18S	Astrophorida	[39]
**Phymaraphiniidae**			
* Exsuperantia*	28S, CO1, 18S	Astrophorida	[14], [36]
**Scleritodermidae**			
* Aciculites*	18S, 28S, CO1	Tetractinellida	[39], [46], [48]
* Microscleroderma*	18S, 28S, EF1alpha, ATPSb-iII	Tetractinellida	[7], [39], [47]
* Scleritoderma*	18S	Tetractinellida	[14]*
**Siphonidiidae**			
* Siphonidium*	18S	Spirophorida	[14]*
**Theonellidae**			
* Discodermia*	18S, CO1, ITS2, 28S	Astrophorida	[14], [36], [38], [39], [42], [45], [46]
* Manihinea*	18S	Astrophorida	[39]
* Theonella*	18S, 28S, CO1	Astrophorida	[14], [36], [38], [39], [46], [48]
**Vetulinidae**			
* Vetulina*	18S, 28S	Sister-group to Spongillida	[14], [38], [39], [45], [46]

Sequences not in GenBank are marked with an asterisk against the corresponding references.

### Aims of this study

This study examines the molecular signatures of 68 lithistid specimens belonging to 12 of the 13 lithistid families, and 21 of the 46 known genera based on new material from different localities worldwide. The study aims to (1) establish a robust molecular phylogeny of lithistids based on independent mitochondrial protein coding (CO1, “Folmer fragment”) and nuclear ribosomal (28S rDNA, partition C1–D2) markers; (2) formally propose the reallocation of all but one lithistid family to their closest relatives, and integrate both molecular and morphological evidence; (3) study the complexity of spicule evolution within lithistid and astrophorid sponges to assess the importance of homoplasy in megascleres and microscleres through a newly constructed morphological character data matrix.

## Materials and Methods

### Taxonomy and Sample datasets

Most of the newly sequenced material (44 out of 68 specimens) was provided by the Queensland Museum Collection (QM) (South Brisbane, Australia) and morphological description of these specimens was published by Schlacher-Hoenlinger et al. (2005) [51]. In addition, 17 specimens from the Western Australian Museum (WAM) (Perth, Australia) were included and identified to genus by one of the authors (AP). Three specimens from French Polynesia were collected by C. Debitus (GW####) and identified to genus by one of the authors (AP) [17]. Four specimens identified by R.W.M. van Soest NCB Naturalis, Leiden, The Netherlands (ZMA POR#####) were also included. Other sequence data used was acquired from GenBank (Table 2).

**Table 2:** Localities of sponge specimens, museum voucher numbers, GB and ENA accession numbers used in this study.

Species	Voucher	GB/ENA Accession Number CO1	GB/ENA Accession Number 28S (C1–D2)	Location
**Family Corallistidae**				
*Herengeria auriculata*	QMG318643		**LN624145**	Norfolk Ridge, Seamount (Pacific Ocean)
*Herengeria auriculata*	QMG318566		**LN624146**	Norfolk Ridge, Introuvable Seamount (Pacific Ocean)
*Herengeria auriculata*	QMG318651		**LN624147**	Norfolk Ridge, Seamount (Pacific Ocean)
*Herengeria auriculata*	QMG318575		**LN624148**	Norfolk Ridge, Eponge Seamount (S-New Caledonia)
*Herengeria vasiformis*	QMG318594		**LN624149**	Norfolk Ridge, Sud-NC Seamount (S-New Caledonia)
*Herengeria vasiformis*	QMG318771		**LN624150**	Norfolk Ridge, Seamount (Pacific Ocean)
*Herengeria* sp.	WAM Z13629		**LN624151**	North West Cape (W-Australia)
*Herengeria* sp.	WAM Z35669	**LN624187**		Jurien Bay (W-Australia)
*Herengeria* sp.	WAM Z35673	**LN624188**		Kalbarri (W-Australia)
*Herengeria* sp.	WAM Z35676	**LN624189**		Zuytdorp (W-Australia)
*Herengeria* sp.	WAM Z35675	**LN624190**		Kalbarri (W-Australia)
*Isabella mirabilis*	QMG318765	**LN624214**	**LN624152**	Norfolk Ridge, Seamount (Pacific Ocean)
*Isabella mirabilis*	QMG318803	**LN624215**	**LN624153**	Norfolk Ridge, Seamount (Pacific Ocean)
*Isabella mirabilis*	QMG318560	**LN624213**		Norfolk Ridge, Seamount (Pacific Ocean)
*Isabella mirabilis*	QMG318737		**LN624154**	Norfolk Ridge, Seamount (Pacific Ocean)
*Neoschrammeniella castrum*	QMG318586	**LN624191**	**LN624155**	Norfolk Ridge, Eponge Seamount (S-New Caledonia)
*Neoschrammeniella norfolki* *	QMG318555		**LN624156**	Norfolk Ridge, Introuvable Seamount (Pacific Ocean)
*Neoschrammeniella norfolki*	QMG317917		**LN624157**	Solomon Islands
*Neophrissospongia* sp.	WAM Z36053		**LN624158**	Adele (W-Australia)
*Neophrissospongia* sp.	WAM Z35946		**LN624159**	Imperieuse Reef (W-Australia)
*Neophrissospongia nolitangere*	MNHN DJV21		AF062602	La Ciotat (France, Mediterranean Sea)
**Family Desmanthidae**				
*Desmanthus incrustans*	QMG325782	**LN624192**		Gulf of Carpentaria (Queensland, Australia)
*Petromica pacifica*	QMG321706	**LN624193**		Eastern end, North West I (Queensland, Australia)
*Petromica pacifica*	QMG320001	**LN624194**		Keppel Island (Queensland, Australia)
*Petromica* sp.	ZMA POR12543	**LN624195**		N of Bird Island, (W-Indian Ocean, Seychelles, Mahé)
**Family Isoraphiniidae**				
*Costifer* sp.	QMG319778	**LN624196**		Solomon Islands
**Family Neopeltidae**				
*Callipelta* sp.	WAM Z12392	**LN624197**		North West Cape (W-Australia)
**Family Macandrewiidae**				
*Macandrewia rigida*	QMG317931		**LN624160**	Solomon Islands
**Family Phymatellidae**				
*Neoaulaxinia* sp.	WAM Z35668		**LN624161**	Perth Canyon (W-Australia)
*Neoaulaxinia* sp.	WAM Z35611		**LN624162**	Two Rocks (W-Australia)
*Neoaulaxinia* sp.	QMG326439	**LN624198**		Cascade Seamount (Tasmania, Australia)
*Neoaulaxinia* sp.	QMG326468	**LN624199**		Cascade Seamount (Tasmania, Australia)
*Neoaulaxinia* sp.	QMG326478	**LN624200**		Cascade Seamount (Tasmania, Australia)
*Neoaulaxinia* sp.	QMG326176	**LN624201**		Cascade Seamount, Huon-slope (Tasmania, Australia)
*Neoaulaxinia* sp.	QMG326476	**LN624202**		Cascade Seamount (Tasmania, Australia)
*Reidispongia coerulea*	QMG318642	**LN624203**	**LN624163**	Norfolk Ridge, Seamount (Pacific Ocean)
*Reidispongia coerulea*	QMG318600		**LN624164**	Norfolk Ridge, Eponge Seamount (S-New Caledonia)
*Reidispongia coerulea*	QMG318563	**LN624204**		Norfolk Ridge, Eponge Seamount (S-New Caledonia)
**Family Pleromidae**				
*Anaderma rancureli*	QMG318561	**LN624205**	**LN624165**	Norfolk Ridge, Bank No1 Seamount (Pacific Ocean)
*Anaderma rancureli*	QMG318821		**LN624166**	Norfolk Ridge, Seamount (Pacific Ocean)
*Anaderma rancureli*	QMG318725		**LN624167**	Norfolk Ridge, Seamount (Pacific Ocean)
*Anaderma rancureli*	QMG318832		**LN624168**	Norfolk Ridge, Seamount (Pacific Ocean)
*Pleroma menoui*	QMG316523		**LN624169**	Norfolk Ridge, Seamount (Pacific Ocean)
*Pleroma menoui*	QMG317900	**LN624206**	**LN624170**	Solomon Islands
*Pleroma menoui*	QMG316513	**LN624207**	**LN624171**	W-Norfolk Ridge, Seamount (Pacific Ocean)
*Pleroma* sp.	WAM Z35947		**LN624172**	Imperieuse Reef, (W-Austarlia)
**Family Scleritodermidae**				
*Aciculites orientalis*	QMG318638		**LN624173**	Norfolk Ridge, Seamount (Pacific Ocean)
*Microscleroderma herdmani*	QMG316621		**LN624174**	Lord Howe Rise, Seamount (Pacific Ocean)
*Microscleroderma* sp.	GW2935		**LN624175**	Ekamako cave, Nuku Hiva (Marquesas Island)
*Microscleroderma* sp.	GW2936		**LN624176**	Ekamako cave, Nuku Hiva (Marquesas Island)
*Microscleroderma* sp.	GW2933		**LN624177**	Tepari cave, Tahiti Iti (Windward, Society Island)
*Scleritoderma camusi*	QMG317903		**LN624178**	Solomon Islands
*Scleritoderma flabelliforme*	QMG318641		**LN624179**	Norfolk Ridge, Seamount (Pacific Ocean)
*Scleritoderma flabelliforme*	QMG318658		**LN624180**	Norfolk Ridge, Seamount (Pacific Ocean)
*Scleritoderma flabelliforme*	QMG318664		**LN624181**	Norfolk Ridge, Seamount (Pacific Ocean)
**Family Siphonidiidae**				
*Siphonidium* sp.	WAM Z36104		**LN624182**	Broome (W-Australia)
**Family Theonellidae**				
*Discodermia polymorpha*			AF062603	La Ciotat (France, Mediterranean Sea)
*Discodermia polymorpha*	ZMBN 85237	HM592686	HM592819	La Ciotat (France, Mediterranean Sea)
*Discodermia proliferans*	G318639		**LN624183**	Norfolk Ridge, Seamount (Pacific Ocean)
*Theonella conica*	UCMPWC1025		HM592818	Near Selapiu Island (Papua New Guinea)
*Theonella mirabilis*	ZMA POR16788	**LN624208**	**LN624184**	N-Cape-Hedo (W-Pacific, Japan, Okinawa)
*Theonella swinhoei*	ZMA POR16637	HM592745	HM592820	Hurghada (Egypt)
*Theonella* sp.	WAM Z35071	**LN624209**	**LN624185**	Point Cloates (W-Australia)
*Theonella* sp.	WAM Z35945		**LN624186**	Imperieuse Reef (W-Australia)
*Theonella* sp.	WAM Z37115	**LN624210**		Dampier Peninsula (W-Australia)
**Family Phymaraphiniidae**				
*Exsuperantia clava*	ZMA POR 21668	HM592730	HM592830	Seamounts south of Azores
**Family Vetulinidae**				
*Vetulina* sp.	WAM Z35842	**LN624211**		Ashmore Reef (W-Australia)
*Vetulina* sp.	WAM Z36103	**LN624212**		Broome (W-Australia)
**Family Geodiidae**				
** Subfamily Geodinae**				
*Geodia gibberosa*	ZMBN 77928	EU442209	FJ717708	Bocas del Toro (Panama, Atlantic)
*Geodia vosmaeri*	ZMBN 85213	HM592722	HM592817	Key Largo, Florida keys, FL (U.S.A.)
*Geodia cydonium*	ZMA POR21652	HM592738	HM592806	Berlengas (Portugal)
*Geodia macandrewii*	ZMBN 77924	EU442198	EU552082	Korsfjord (Western Norway)
*Geodia baretti*	ZMBN 77922	EU442194	EU552080	Korsfjord (Western Norway)
*Geodia baretti*	ZMBN 85202	HM592720	HM592809	Hebrides Islands (Scotland)
*Geodia megastrella*	ZMA POR21654	HM592731		Seamounts south of Azores
** Subfamily Erylinae**				
*Pachymatisma johnstonia*	MNHN DCL4015		AF062601	Roscoff (France)
*Pachymatisma johnstonia*	ZMA POR21442	EF564338	HM592832	Berlengas Islands (Portugal)
*Pachymatisma johnstonia*	ZMA POR20348a	EF564330		Minggulay reef (Scotland)
*Erylus discophorus*	ZMA POR21716	HM592692	HM592822	Piran (Slovenia)
*Erylus granularis*	ZMA POR21656	HM592729	HM592827	Seamounts south of Azores
*Erylus topsenti*	ZMA POR21657	HM592733	HM592831	Seamounts south of Azores
**Family Ancorinidae**				
*Ancorina* sp.	ZMA POR21660	HM592744	HM592785	Gorringe Bank
*Ecionemia megastylifera*	ZMBN 81782	FJ711642	FJ711648	Bocas del Toro (Panama, Caribbean)
*Ecionemia robusta*	S1018	HM592724	HM592802	Investigator group Island (South Australia)
*Rhabdastrella cordata*	S1026	HM592727	HM592813	Investigator group Island (South Australia)
*Stelletta tuberosa*	ZMA POR21665	HM592735	HM592799	Seamounts south of Azores
*Stelletta lactea*	Mc4945	HM592752	HM592795	Strangford Lough (Northern Ireland)
*Stelletta clarella*	ZMA POR21673	HM592736	HM592797	Monterey Bay, CA (U.S.A.)
**Family Calthropellidae**				
*Calthropella geodioides*	ZMA POR21667	HM592734	HM592825	Seamounts south of Azores
*Calthropella geodioides*	MNHN DCL4076	HM592705	HM592826	Off Cape S. Maria di Leuca (Southern Italy)
**Family Pachastrellidae**				
*Characella pachastrelloides*	ZMA POR20375	HM592749	HM592781	Mingulay Reef, Scotland (United Kingdom)
*Pachastrella nodulosa*	ZMBN 85227	HM592698	HM592775	Korsfjord (Western Norway)
*Poecillastra compressa*	MNHN DCL4072	HM592714	AF062599	Banc de l’Esquine (France, Mediterranean Sea)
*Thenea abyssorum*	ZMBN 85228	HM592712	HM592770	Greenland Sea
*Thenea levis*	ZMBN 85230	HM592717	HM592765	Off Korsfjord (Western Norway)
*Thenea schmidti*	ZMA POR18036	HM592737		Gulf of Cadiz
*Triptolemma intextum*	MNHN DCL4080	HM592710	HM592777	Off Cape S. Maria di Leuca (Southern Italy)
*Vulcanella aberrans*	ZMBN 80959	HM592699	HM592758	Sotbakken (Northern Norway)
*Vulcanella gracilis*	MNHN DCL4082	HM592704	HM592760	Off Cape S. Maria di Leuca (Southern Italy)
**Family Alectonidae**				
*Alectona millari*	ZMBN 85238	HM592670		Sotbakken (Northern Norway)
**Family Tetillidae**				
*Cinachyrella alloclada*	DH S271 = TAU25617		JX177935	
*Cinachyrella apion*	ZMBN 81789	HM592667	HM592753	Key Largo, FL (U.S.A.)
*Cinachyrella kuekenthali*		EF519603		
*Cinachyrella schulzei*	G320636	HM032745		
*Cinachyrella levantinensis*	DH S124 = TAU 25618		JX177938	
*Cinachyrella levantinensis*	TAU 25529		JX177939	
*Cinachyrella levantinensis*	MHNM 16194		JX177941	
*Cinachyra barbata*	NIWA 28877		JX177950	
*Cinachyra antarctica*	NIWA28957		JX177949	
*Craniella* cf. *leptoderma*	G315031		JX177942	
*Craniella* cf. *leptoderma*	NIWA 36097		JX177944	
*Craniella* cf. *leptoderma*	NIWA 28524		JX177945	
*Craniella* cf. *leptoderma*	NIWA 28496		JX177946	
*Craniella* cf. *leptoderma*	NIWA 28507		JX177943	
*Craniella cranium*	ZMBN 85239	HM592669		Korsfjord (Western Norway)
*Craniella zetlandica*		HM032751		
*Craniella* sp.	ZMBN 85240	HM592668		Korsfjord (Western Norway)
*Fangophilina* sp.	NIWA 28614		JX177952	
*Fangophilina* sp.	NIWA 28586		JX177953	
*Paratetilla bacca*	G306342		JX177927	
*Amphitethya* cf. *microsigma*	SAM S1189		JX177929	
**Family Agelasidae**				
*Astrosclera willeyana*	UCMPWC 1070	AY561969		
*Agelas dispar*		EF519546		
*Axinella corrugate*		NC006894		
*Prosuberites laughlini*	UCMPWC 875	AY561960		Caribbean
**Family Dictyonellidae**				
*Dictyonella* sp.		AM498649		
*Acanthella acuta*	Mc7160	HQ379408		Mediterranean
*Phakellia ventilabrum*	Mc4248	HQ379409		Scotland
**Family Spongillidae**				
*Baikalospongia bacillifera*		EU000570		
*Ephydatia fluviatilis*	ZMB Por12658	DQ167174		
*Lubomirskia baicalensis*	ZMB Por12654	DQ167169		
*Eunapius fragilis*		AJ843882		
*Spongilla lacustris*		AJ843883		
*Spongilla lacustris*		EU000572		
*Pachydictyum globosum*	ZMB Por12649	DQ167177		
**Family Tethyidae/Hemiasterellidae**				
*Tethya aurantium*		EF584565		Mediterranean
*Tethya citrina*	Mc5113	HQ379427		Wales
*Adreus fascicularis*	Mc4559	HQ379428		English Channel
**Family Clionaidae**				
*Pione vastifica*		EF519665		
*Cliona celata*		EF519608		
**Family Polymastiidae**				
*Polymastia janeirensis*		EU076813		Brazil
*Sphaerotylus* sp.	Mc4236	HQ379425		Ireland
**Family Desmacellidae**				
*Biemna fistulosa*	TAU 25197	AM076982		
*Neofibularia nolitangere*		EF519653		Caribbean
**Family Scopalinidae**				
*Svenzea zeai*		EF519682		Caribbean
*Scopalina lophyropoda*	Mc4217	HQ379411		Mediterranean
**Family Poecilosclerida**				
*Crambe crambe*		AF526297		
*Crambe crambe*		AF526298		
*Monanchora arbuscula*		EF519645		
*Clathrina oxeota*		EF519605		
*Acantheurypon pilosella*	Mc7607	JF440337		Ireland
*Crella elegans*		JF440338		Mediterranean
*Neopodospongia* sp.		JF440339		Ireland
*Mycale laxissima*		EF519649		Caribbean
*Tedania ignis*		DQ133896		Panama
**Family Suberitidae & Halichondriidae**				
*Protosuberites* sp.	POR14649	AY561979		
*Hymeniacidon heliophila*		EU076812		Brazil
*Suberites ficus*		AJ843891		
*Suberites domuncula*		AM690374		Adriatic Sea
*Halichondria melanodocia*		EF519617		Caribbean
*Topsentia ophiraphidites*		EU237482		
**Family Raspailiidae**				
*Raspailia ramose*	Mc4024	HQ379417		Ireland
*Raspailia hispida*	Mc3597	HQ379416		Ireland
*Pandaros acanthifolium*		EF519662		Caribbean
*Raspaciona aculeata*	Mc7159	HQ379415		Mediterranean
*Eurypon clavigerum*	Mc4992	HQ379413		Ireland
*Ectyoplasia ferox*		EF519612		Caribbean
*Endectyon delaubenfelsi*	Mc4527	HQ379412		English Channel
*Tethyspira spinosa*	Mc4641	HQ379418		Ireland
**Family Stelligeridae**				
*Stelligera rigida*	Mc4357	HQ379420		Scotland
*Stelligera stuposa*	Mc4330	HQ379421		Scotland
*Paratimea constellata*	Mc4323	HQ379419		Scotland
*Halicnemia patera*	Mc5427	HQ379422		Ireland
*Halicnemia verticillata*	Mc5018	HQ379414		Ireland
**Family Axinellidae**				
*Dragmacidon reticulatum*		AJ843894		
*Axinella infundibuliformis*	Mc4438	HQ379410		Scotland
**Outgroups**				
*Aplysina aerophoba*		EF043371		
*Hexadella pruvoti*		FN667709		
*Pleraplysilla* sp.		EF519667		
*Verongula rigida*		EF519695		
*Axinella damicornis*			AF062605	
*Halichondria panicea*			AF062607	

New sequences from this study are highlighted in bold.

*Fragment of holotype.

Abbreviations:

ZMA POR: Zoölogisch Museum van de Universiteit van Amsterdam.

QMG: Queensland Museum, Brisbane, Australia.

WAM: West Australian Museum.

ZMB Por: Museum for Natural History Humboldt Universität.

Mc: National Museums, Northern Ireland, Holywood.

UCMPW: University of California Museum of Paleontology, Berkeley, CA.

GW: Molecular Paleo- & Geobiology Munich, Germany.

ZMBN: Zoologisk Museum Bergen.

S/SAM: South Australian Museum, Adelaide.

MNHN: Muséum National d’Histoire Naturelle, Paris.

TAU: Steinhardt National Collection of Natural History, Zoological Museum at Tel Aviv University, Israel.

DH: Lab collections of Amir Szitenberg, Department of Zoology, Israel.

NIWA: National Institute of Water and Atmospheric Research, New Zealand.

A list of specimens used in this study with their corresponding voucher number, locality, GenBank (GB) and European Nucleotide Archive (ENA) accession numbers are given in Tab. 2. New sequences from this study are available from the ENA under the accession numbers LN624145-LN624186 (http://www.ebi.ac.uk/ena/data/view/LN624145-LN624186) for the 28S gene and LN624187–LN624215 (http://www.ebi.ac.uk/ena/data/view/LN624187-LN624215) for the CO1 gene. Additionally, all CO1 barcoding sequences and additional specimen-specific data are available at the Sponge Barcoding Database (SBD) (http://www.spongebarcoding.org/) (record numbers 1122 to 1150).

### DNA extraction, amplification and sequencing

Genomic DNA was extracted using a modified [52] PALL-plate based extraction method [53] with an increased amount of tissue and twice the amount of lysis mix. In order to avoid any clogging of the membrane an additional centrifugation step was added just before transferring the lysate to the PALL-plates. For some specimens, where only little tissue was available, DNA was extracted using the NucleoSpinTissue Kit (Macherey-Nagel, Düren, Germany), following the standard protocol with an additional centrifugation step before pipetting the lysate to the Spin Column. To quantify the amount of isolated genomic DNA, a NanoDrop 1000 Spectrophotometer (Thermo Scientific) was used. The following two unlinked genes were amplified for this study: The standard DNA barcoding fragment (cytochrome oxidase subunit 1, partial; 659 bp) using the primers dgLCO1490 and dgHCO2198 [54] and following the protocol: 95°C, 3 minutes; (95°C, 30 seconds; 40–43°C, 20 seconds; 72°C, 1 minute) ×34 cycles; 72°C, 5 minutes. The 28S rDNA (partition C1–D2, 768–832 bp) was studied using the forward C1’ASTR [55] and the reverse universal D2 primers [56], with the following PCR settings of 95°C, 3 minutes; (95°C, 30 seconds; 56–59°C, 45 seconds; 72°C, 1 minute) ×35 cycles; 72°C, 5 minutes. PCR products were cleaned for sequencing using a standard ammonium acetate-ethanol precipitation [57]. Sequencing reactions of both strands with the same primers were carried out using BigDye Terminator v3.1 (Applied Biosystems, Forster City, CA, USA) and analyzed on an ABI 3730 Genetic Analyzer at the Sequencing Service of the Department of Biology, LMU München. The raw trace files where post-processed by base-calling, trimming and contig assembly in CodonCode Aligner v.3.7.1.1 (CodonCode Corporation) and subsequently checked by eye. The sponge origin of the sequences was evaluated by BLAST searches against NCBI GenBank (http//blast.ncbi.nlm.nih.gov).

### Phylogenetic reconstruction

#### Sequence alignments and outgroup choice

Newly generated sequences as well as downloaded GenBank sequences of the CO1 and 28S gene were separately aligned with Muscle (v.3.6) [58] as incorporated in SeaView [59]. Alignments were subsequently controlled by eye. Saturation of both markers (CO1 and 28S) was evaluated using Xia’s test [60] as implemented in DAMBE v5.1.5 [61]. This entrophy-based index estimates a substitution saturation index (Iss) and compares it to a critical substitution saturation index (Iss.c). As both datasets (CO1 and 28S) were too different from each other with respect to taxon sampling and sequencing success of the CO1 gene region to be merged, analyses were done separately for each gene region. As ‘Lithistida’ is a polyphyletic group, a wide range of sequences from GenBank from Heteroscleromorpha families were added to the CO1 dataset to yield a representative adequate taxon set from the latest classification of Demospongiae according to Morrow et al. (2012) [49]. For the CO1 dataset, sequences of the subclasses Verongimorpha and Keratosa were chosen as outgroups, as these subclasses have shown shorter branch lengths than Haplosclerida, the sister group of Heteroscleromorpha [62]. *Axinella damicornis* and *Halichondria panicea* were chosen as outgroups for our 28S rDNA (C1–D2 partition). The alignments used in this study, as well as the morphological data matrix (see below) in Nexus format, are freely available at OpenDataLMU (http://dx.doi.org/10.5282/ubm/data.66).

#### Phylogenetic analyses

For Bayesian phylogenetic analyses we used the parallel version of MrBayes v.3.1.2 [63] on a Linux cluster under the most general GTR+G+I model, as possible overparameterization does not appear to have a negative effect on the results [64]. Analyses were run in two concurrent runs of four Metropolis-coupled Markov-chains (MCMC) for 100,000,000 generations or stopped when the average standard deviation of split frequencies decreased below 0.01. The first 25% of the sampled trees were discarded for further analysis as burn-in. In both datasets, Maximum Likelihood (ML) bootstrap analyses (1,000 replicates) were also performed under the GTRGAMMAI nucleotide evolution model using raxmlGUI v.1.3 [65]. Tree topologies from Bayesian and ML analyses were compared and visualized using TreeGraph2 [66].

### Morphological Analyses

In order to investigate spicule evolution of megascleres and microscleres within the Astrophorida/lithistids we used Mesquite v2.75 [67]. We designed a new character data matrix for lithistid sponges from our own observed data, and amended it with carefully selected data from another study (Cárdenas et al. 2011), representing the smaller part of the whole matrix. In total the final matrix consists of 69 taxa and 35 characters coded as 1 for present or 0 for absent (see S1 Table and S1 File). For tracing characters over the imported molecular Bayesian tree and testing the homoplasy within lithistids and astrophorids the parsimony ancestral state reconstruction method was used under the unordered state assumption.

## Results

### Comparison of both gene trees

**Figure 5:**
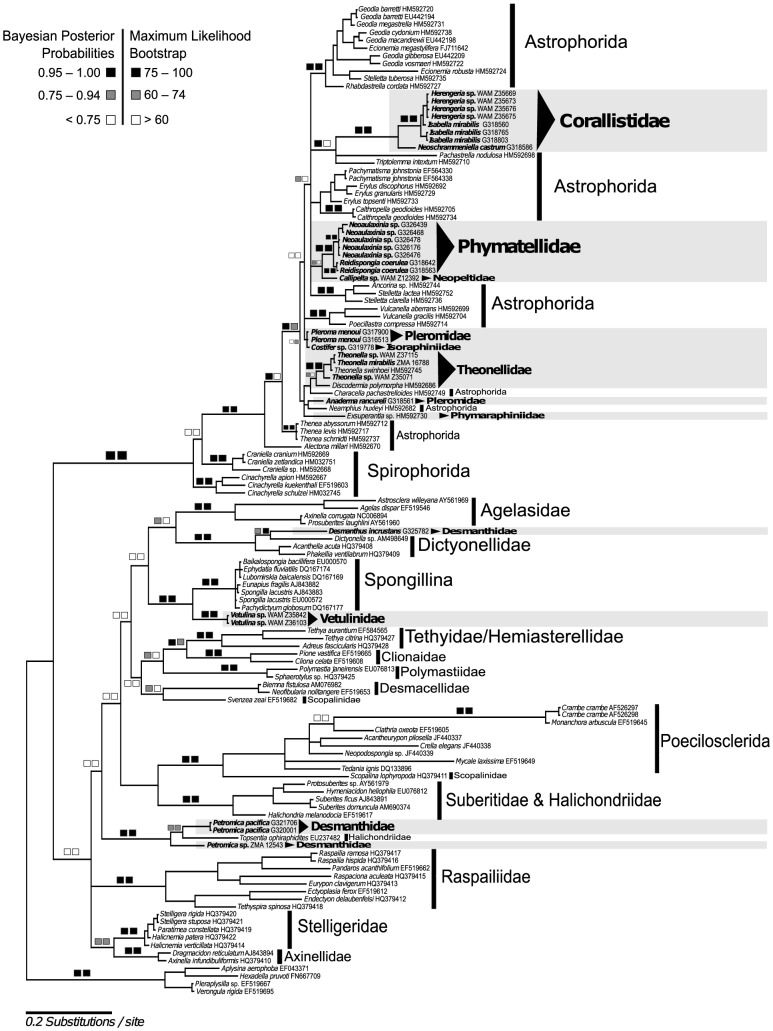
Bayesian Inference (MrBayes, GTR+I+G model) phylogeny of a representative selection of demosponge taxa based on CO1. The maximum likelihood (RAxML) tree is congruent. Squares represent node supports. Black squares: PP = 0.95–1.00, BP = 75–100. Dark gray squares: PP = 0.75–0.94, BP = 60–74. White squares: PP<0.75, BP<60. Black triangle indicates lithistid families. Numbers behind taxon names are either voucher numbers or GenBank accession numbers. Self-generated sequences are in bold.

Both molecular markers were not significantly saturated, as the Iss.c (0.801) was significantly higher than the observed Iss (0.286), therefore, both markers are suitable for conducting phylogenetic analyses with lithistid demosponges. The CO1 gene tree (Fig. 5) was used to resolve the classification of lithistids sequenced here with respect to other major demosponge groups. The resulting data matrix from the CO1 gene comprises 121 taxa. From 121 taxa, 31 are lithistids, from which 29 are represented by *de novo*-generated sequences and two (*Theonella swinhoei* and *Exsuperantia* sp.) obtained from GenBank. The 28S rDNA data matrix included 94 taxa, of which 48 are lithistids (43 *de novo*-generated sequences and five sequences from GenBank). An overview of sequencing success is given in Table 3. Bayesian inference and Maximum Likelihood topologies are congruent in both analyses.

**Table 3:** Summary of taxonomic changes from our present study and previous studies.

Lithistid taxa	Gene Region	Reallocation	References
**Azoricidae**			
* Leiodermatium*	18S	Tetractinellida	[14]*
**Corallistidae**			
* Corallistes*	18S, 28S, CO1, ITS	Astrophorida	[39], [45], [46]
*** Neophrissospongia***	18S, **28S**, CO1	**Astrophorida**	[36], [39], **PS**
*** Herengeria***	**28S, CO1**	**Astrophorida**	**PS**
*** Neoschrammeniella***	**28S, CO1**	**Astrophorida**	**PS**
*** Isabella***	**28S, CO1**		**PS**
**Desmanthidae**			
* Desmanthus*	28S, 18S, **CO1**	**Dictyonellidae**	[39], [49], **PS**
* Petromica*	18S, **CO1**	**Halichondriidae**	[14], [39], **PS**
**Isoraphiniidae**			
*** Costifer***	**CO1**	**Astrophorida**	**PS**
**Macandrewiidae**			
*** Macandrewia***	**28S**	**Astrophorida**	**PS**
**Neopeltidae**			
*** Callipelta***	28S, 18S, **CO1**	**Astrophorida**	[39], [50], **PS**
* Homophymia*	18S	Astrophorida	[39]
**Phymaraphiniidae**			
* Exsuperantia*	28S, CO1, 18S	Astrophorida	[14], [36]
**Phymatellidae**			
*** Neoaulaxinia***	**28S, CO1**	**Astrophorida**	**PS**
*** Reidispongia***	**28S, CO1**	**Astrophorida**	**PS**
**Pleromidae**			
*** Anaderma***	**28S, CO1**	**Astrophorida**	**PS**
*** Pleroma***	**28S, CO1**	**Astrophorida**	**PS**
**Scleritodermidae**			
*** Aciculites***	18S, **28S**, CO1	**Tetractinellida**	[39], [46], [48], **PS**
*** Microscleroderma***	18S, **28S**, EF1alpha, ATPb	**Tetractinellida**	[7], [39], [47], **PS**
*** Scleritoderma***	18S, **28S**	**Tetractinellida**	[14]*, **PS**
**Siphonidiidae**			
*** Siphonidium***	18S, **28S**	Tetractinellida	[14]*, **PS**
**Theonellidae**			
* Discodermia*	18S, CO1, ITS2, **28S**	**Astrophorida**	[14], [36], [38], [39], [42], [45], [46], **PS**
* Manihinea*	18S	Astrophorida	[39]
*** Theonella***	18S, 28S, **CO1**	**Astrophorida**	[14], [36], [38], [39], [46], [48], **PS**
**Vetulinidae**			
*** Vetulina***	18S, 28S, **CO1**	**Sister-group to Spongillida**	[14], [38], [39], [45], [46], **PS**

Self-generated sequences, proposed reallocation from our data are marked in bold. PS for Present study.

### Intra-family relationships of lithistid sponges in relation to other demosponges

**Figure 6:**
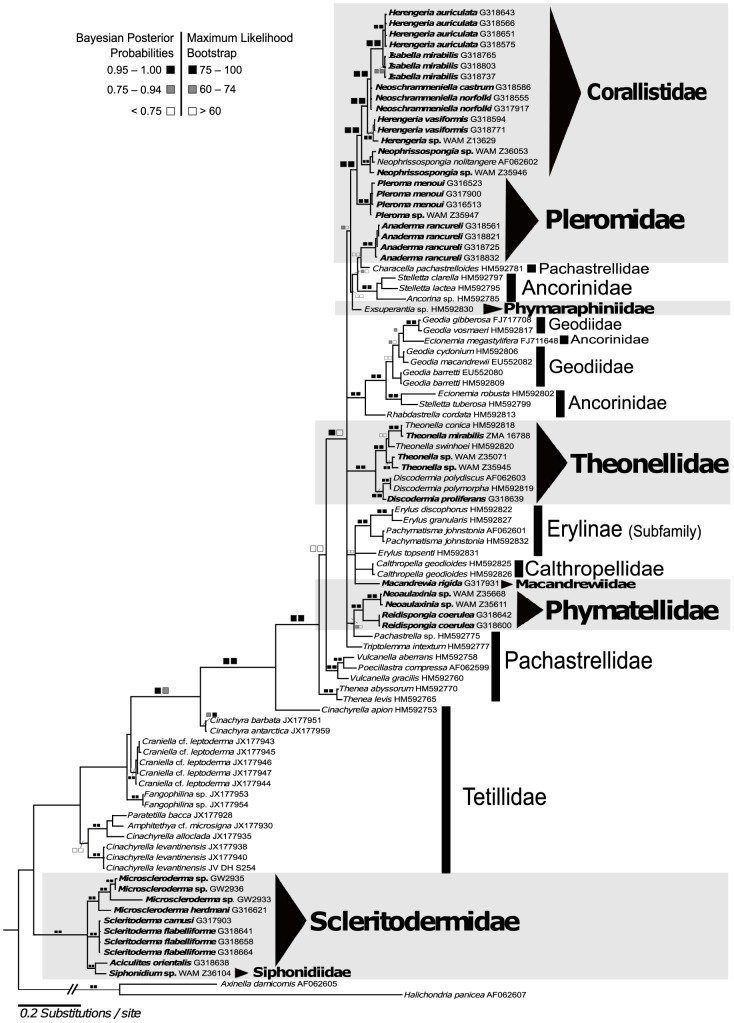
Bayesian Inference (MrBayes, GTR+I+G model) phylogeny of a representative selection of demosponge taxa based on 28S rDNA (partition C1–D2). The maximum likelihood (RAxML) tree is congruent. Squares represent node supports. Black squares: PP = 0.95–1.00, BP = 75–100. Dark gray squares: PP = 0.75–0.94, BP = 60–74. White squares: PP<0.75, BP<60. Black triangle indicates lithistid families. Numbers behind taxon names are either voucher numbers or GenBank accession numbers. Self-generated sequences are in bold.

Based on both CO1 (Fig. 5) and 28S rDNA (Fig. 6) gene trees, the families Corallistidae, Pleromidae, Theonellidae, Phymatellidae, Phymaraphiniidae, Neopeltidae and Isoraphiniidae are nested within the Astrophorida. Additionally, the family Macandrewiidae is supported by our 28S rDNA dataset to also belong to the Astrophorida, indicating that 8 out of 13 families belong to the Astrophorida. A strongly supported clade (bootstrap: 100%, posterior probability 1.0) as a result of our 28S rRNA analysis (no CO1 data have been obtained yet) containing the family Scleritodermidae represented by the three genera (*Microscleroderma, Scleritoderma* and *Aciculites*), as well as the family Siphonidiidae (*Siphonidium* sp.) are the sister group to Tetillidae/Astrophorida. The family Desmanthidae (genera *Desmanthus* and *Petromica*) is recovered as polyphyletic. The genus *Petromica* forms a highly supported clade (bootstrap of 99% and posterior probabilities 1.00) with the halichondriid *Topsentia ophiraphidites*. *Desmanthus incrustans* is sister to the single specimen of *Dictyonella* sp. (bootstrap: 84%, posterior probability: 0.75) of the family Dictyonellidae. This clade shows high bootstrap (99%) and posterior probability (1.00) support values. The monogeneric lithistid family Vetulinidae forms a highly supported clade with Spongillina (96% bootstrap and 1.00 posterior probability).

### Phylogenetic relationships of lithistid sponges within the Tetractinellidae

#### Family Corallistidae

The family Corallistidae is monophyletic in both CO1 (Fig. 5) and 28S rDNA (Fig. 6) gene trees. The genus *Herengeria* is polyphyletic, *Isabella* is not monophyletic, and *Neophrissospongia* is monophyletic. All these clades are strongly supported. The 28S gene analysis does not resolve the genus *Neoschrammeniella* as monophyletic. However, both gene trees indicate a sister group relationship of *Neoschrammeniella castrum* and *Neoschrammeniella norfolki* to the *Herengeria*/*Isabella* clade, with high support values (bootstrap of 82% and posterior probabilities of 0.99). Species of *Herengeria auriculata* are sister to *Isabella mirabilis*, which is also highly supported in both CO1 and 28S rDNA gene trees. The genus *Neophrissospongia* represents a sister clade to the genera *Herengeria*, *Neoschrammeniella* and *Isabella*.

#### Family Pleromidae

The family Pleromidae is polyphyletic and is represented by the genera *Pleroma* and *Anaderma*. The genus *Anaderma* seems to be related to *Characella pachastrelloides incertae sedis, sensu* Cárdenas et al. (2011, 2012) [36], [68], however this is not supported by posterior probabilities or bootstrap values. In contrast the genus *Pleroma* is recovered as the sister to the Corallistidae, with strong support (bootstrap of 84% and posterior probability of 1.00).

#### Family Theonellidae

The family Theonellidae is monophyletic and contains the genera *Theonella* and *Discodermia.* Both genera are monophyletic and form a sister group to each other. All nodes are highly supported. The exact position within the Astrophorida however, remains unclear due to low resolution within the gene trees.

#### Family Phymatellidae

Phymatellidae is monophyletic. *Neoaulaxinia* and *Reidispongia* are highly supported to be sister taxa in both gene trees. The CO1 gene tree shows a close relationship of Phymatellidae to the lithistid genus *Callipelta* (Neopeltidae), however this relationship is only moderately supported by a posterior probability of 0.78 and not supported with bootstrap. In contrast the 28S rDNA gene tree shows *Neoaulaxinia* and *Reidispongia* close to *Pachastrella* sp. from the family Pachastrellidae. This finding has a posterior probability of 0.93 and not supported by bootstrap.

#### Families Phymaraphiniidae, Macandrewiidae, Isoraphiniidae and Neopeltidae

The species *Exsuperantia* sp. (family Phymaraphiniidae), *Macandrewia rigida* (family Macandrewiidae), *Costifer* sp. (family Isoraphiniidae) and *Callipelta* sp. (family Neopeltidae), only represented by a single taxon each, clearly group within the Astrophorida. However, the low resolution within both gene trees makes the inference of a clear relationship to other lithistid or astrophorid clades impossible.

#### Family Scleritodermidae and Siphonidiidae

The monophyly of Tetillidae as suggested by Szitzenberg et al. 2013 [69] could not be corroborated in any of our analyses, independently of whether the lithistid families Scleritodermidae and Siphonidiidae were included (28S rDNA gene tree) or not (CO1 gene tree). Scleritodermidae is monophyletic and is represented in the 28S rDNA gene tree with the genera *Microscleroderma*, *Scleritoderma* and *Aciculites.* The genera *Microscleroderma* and *Scleritoderma* are sister groups, while *Aciculites* (Scleritodermidae) group together with *Siphonidium* sp. (Siphonidiidae). All these nodes are highly supported.

### Parsimony reconstruction of ancestral states

**Figure 7:**
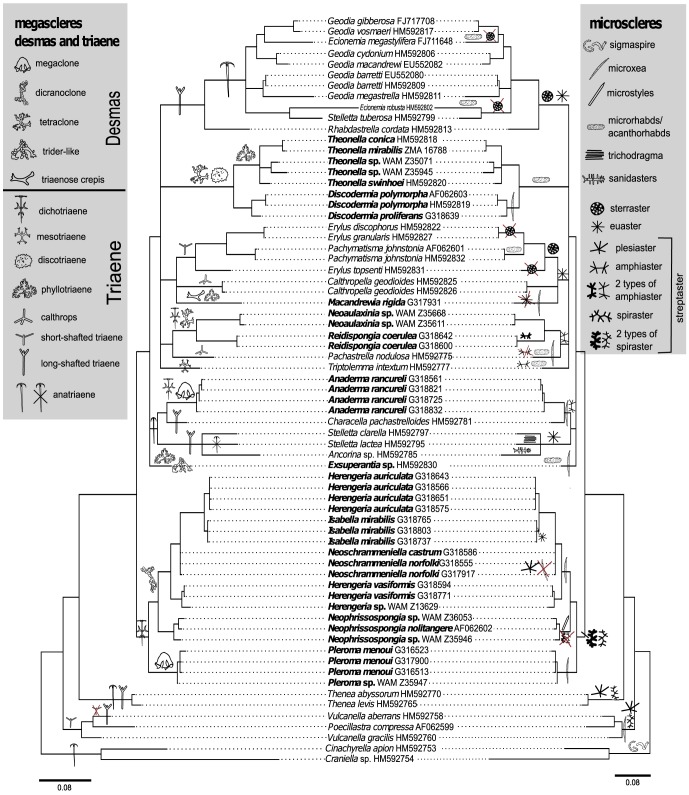
Parsimony ancestral state reconstruction of mega- and microscleres mapped on an imported modified molecular Bayesian Inference 28S rDNA (partition C1–D2) gene tree from Fig. 6 in Mesquite v.2.75. The phylograms represent the presents or absents of megascleres (left) and microscleres (right). Numbers behind taxon names are either voucher numbers or GenBank accession numbers.

A summary of the parsimony reconstruction of possible ancestral states for megascleres and microscleres composed of homologous characters is given in Fig. 7. These were derived from precise morphological descriptions from the literature and new observations in this study. For some taxa, such as *Isabella mirabilis* and *Neoschrammeniella norfolki*, however, there could be difficulties interpreting whether character states of the various streptaster microscleres (spiraster/amphiaster/plesiaster) [51] were homologs or analogs. In this case we followed the definition of streptasters *sensu* Sollas (1888), where amphiasters bear some analogy to spirasters as they are only differentiated in the shaft, which could be either straight ( = amphiaster, see Fig. 4G) or spiral ( = spiraster see Fig. 4I,J). In addition, definitions of other streptasters, like plesiasters and metasters were used as described in the study of Cárdenas et al. 2012 [68]. This produced a total number of 13 megascleres (five different desmas and eight different triaenes) and 13 microscleres (Fig. 7). Our results show possible multiple convergences of megascleres and microscleres. These data indicate that megaclone desmas could have evolved two times independently in *Pleroma* and *Anaderma* and tetraclone desmas could have developed twice independently in the families Theonellidae and Phymatellidae. By comparison, dicranoclone desmas possibly evolved only once in the family Corallistidae, and desmas of triaenose crepis (*Exsuperantia* sp.) and trider-like desmas (*Macandrewia rigida*) also possibly developed only once. Dichotriaenes could have evolved three times independently in the families Phymatellidae and Corallistidae as well as in the genus *Anaderma*. Mesotriaenes (*Triptolemma intextum)* and discotriaenes (Theonellidae) probably only appeared once. Phyllotriaenes could have evolved at least three times independently in the genus *Theonella*, *Macandrewia* and *Exsuperantia*. Anatriaenes may have evolved four times independently in different astrophorid and lithistid groups and were lost in some taxa (e.g. *Stelletta lactea*). Long and short-shafted triaenes were probably lost several times independently in many different astrophorid genera. Calthrops could have appeared at least twice independently according to this dataset (*Pachastrella nodulosa* and *Calthropella geodioides*). Our analysis also indicates a high potential of convergent spicule evolution and numerous secondary losses within most microscleres including amphiasters, spirasters, plesiasters, microxeas, euasters, sterrasters and microrhabds. This mapping indicates that secondary losses are four times more frequent in microscleres than in megascleres.

## Discussion

### Phylogeny of lithistids compared with previous molecular and morphological studies

From molecular phylogenetic analysis of 68 lithistid demosponges (the largest lithistid taxon sampling to date), our study has showed the complexity of spicule evolution within the polyphyletic ‘order Lithistida’. Previously Burton (1929) and de Laubenfels (1936) had suggested the affiliation of triaene-bearing lithistids to the Astrophorida, subsequently affirmed by Lévi (1973) and accepted by Pisera & Lévi (2002) (see Tab.1). Similarly, previous data from different gene regions suggested that the four families Corallistidae, Neopeltidae, Phymaraphiniidae and Theonellidae, including the eight genera (*Corallistes, Neophrissospongia, Callipelta, Homophymia, Exsuperantia, Discodermia, Manihinea,* and *Theonella*) all belonged to the Astrophorida (for references see Tab.1). Our study corroborates all these findings and additionally provides evidence that *Herengeria*, *Isabella* and *Neoschrammeniella* (Corallistidae) should also be included in Astrophorida. Previously, the assignment of the four triaene-bearing lithistid families Isoraphiniidae, Macandrewiidae, Phymatellidae and Pleromidae to the Astrophorida was based only on morphological observations [36], [39], and is now confirmed based with molecular data. Molecular analyses undertaken in the present study corroborated these hypotheses for the first time, affirming the relationship of Pleromidae (*Pleroma*, *Anaderma*), Phymatellidae (*Neoaulaxinia*, *Reidispongia*), Isoraphiniidae (*Costifer*) and Macandrewiidae (*Macandrewia*) to the Astrophorida.

Based on spicule morphology Burton (1929) suggested a close relationship of the lithistid genera *Microscleroderma* and *Scleritoderma* (Scleritodermidae), both characterized by rhizoclone desmas, to the spirophorid family Tetillidae due to the possession of similar microscleres (sigmaspires). He also included the rhizoclone desma-bearing genus *Leiodermatium* (Azoricidae) in this group, which lacks sigmaspires. Later morphological observations by de Laubenfels (1936) assigned *Microscleroderma* to the Poecilosclerida. In *Systema Porifera* [70], [71], adopting a conservative taxonomy approach, rhizomorine lithistids were divided into two families: Azoricidae (*Leiodermatium* and *Jereicopsis*) and Scleritodermidae (*Aciculites*, *Amphibleptula*, *Microscleroderma*, *Scleritoderma* and *Setidium*), based on the presence or absence of certain microscleres [70]. Previous molecular phylogenies had suggested a close relationship of the genera *Leiodermatium* (Azoricidae) [35], *Aciculites*, *Microscleroderma* and *Scleritoderma* (Scleritodermidae) [7], [14], [39], [46], [47], [72] to the Tetractinellida. However, the exact relationships to either Astrophorida or Spirophorida remained uncertain. Our 28S rDNA results revealed a highly supported monophyletic clade of Scleritodermidae+Siphonidiidae, which was also partly observed by Redmond et al. (2013) [39]. Conversely, the monophyly of Tetillidae [69] could not be confirmed from any of our analyses, independently of whether or not Scleritodermidae and/or Siphonidiidae were included. This result is also similar to the findings of Redmond et al. (2013) [39]. The clade Scleritodermidae+Siphonidiidae neither belongs to Astrophorida nor to spirophorids, but instead it shows a sister group relationship to the Astrophorida+Spirophorida clade. However, it should be mentioned here that other families of the order Spirophorida (Samidae and Spirasigmidae) are missing in our analysis and thus the exact classification of this clade is still in need of further investigations. Further, the homology and/or convergence of sigmatose microscleres still remains unclear and need further investigations. Our molecular findings that *Aciculites orientalis* (Scleritodermidae) is the sister to *Siponidium* sp. (Siphonidiidae) is supported by morphological observations, where *Aciculites* has relatively more tuberculate rhizoclone desmas and *Siphonidium* has thorny and spined rhizoclone desmas, suggesting that both rhizoclone desmas are analogs and probably belong to different desma categories.

#### Family Vetulinidae Lendenfeld, 1903

Vetulinidae is represented by one genus and species (*Vetulina stalactites*). It is only known from the Caribbean (Barbados) and morphologically characterized by sphaeroclone desmas and the absence of ectosomal spicules and microscleres [73]. Based on morphological observations Van Soest & Stentoft (1988) [74] and Gruber (1993) [75] suggested a close relationship of *Vetulina* to the genera *Siphonidium* (Siphonidiidae) and *Leiodermatium* (Azoricidae). However, Pisera & Lévi (2002) [73] indicated these were weak assumptions and noted the occurrence of uniaxial or polyaxial sphaeroclone or astroclone-like desmas – not observed in any other lithistid or non-lithistid demosponges. Molecular investigations using different markers and fragments (18S and 28S rDNA, see also Tab.1) indicated with strong support the sister group relationship of *Vetulina* to freshwater sponges (Spongillida). Our study strongly confirms and corroborates these findings, for the first time using the mitochondrial CO1 gene. Morphologically, Spongillida differ from *Vetulina* by the presence of microscleres, megascleres and gemmoscleres and absence of sphaeroclone desmas [76]. One explanation could be that Spongillida lost its possession of sphaeroclone desmas, as this process seems more phylogenetically parsimonious than the evolution of new desmas. This discrepancy of morphological versus molecular data remains unresolved at present and needs further attention. As ‘Lithistida’ is no longer an accepted ordinal taxon, and the genus *Vetulina* cannot be assigned to any other existing order of the Demospongiae (as well as their morphological differences to the Spongillida), lead us to the taxonomic action to resurrect Sphaerocladina for Vetulina, based on the existing paleontological concept of Sphaerocladina. Firstly, *Vetulina* has probably been separated from the order Spongillida for a long evolutionary time. Secondly, *Vetulina* has an unequivocally long and continuous history dating back to the Middle Jurassic to the present through the known fossil record [77]. Thirdly, in this particular case there is no reason to create a new higher taxon for a Recent genus when the taxonomic concept is otherwise identical to the continuous palaeontological concept of Sphaerocladina. The taxon Sphaerocladina Schrammen 1924 was first used as a Suborder to include fossil sponges with sphaeroclonar desmas, like those in *Vetulina.*

#### Family Desmanthidae Topsent, 1894

The family Desmanthidae comprises four genera: *Paradesmanthus* Pisera & Lévi, 2002, *Sulcastrella* Schmidt, 1879, *Desmanthus* Topsent, 1894 and *Petromica* Topsent, 1898. They are encrusting sponges with branching monocrepidial desmas. Ectosomal microscleres (sanidaster-like) are only found in the genus *Paradesmanthus*. Burton (1929) and de Laubenfels (1936) had already noted the similarity of these characters to other non-lithistid demosponges, and assumed a close relationship to the Halichondrida. Morphologically, desmas of *Petromica* are different from those found in other genera of this family, which would support the polyphyly of this family. Morphological descriptions of *Lithobubaris* ( = *Sulcastrella*) confirm the close relationship of *Desmanthus*, *Sulcastrella* and *Paradesmanthus* to the bubarid genera *Monocrepidium* and *Bubaris* [78]. Pisera & Lévi (2002) [79] acknowledged the resemblance of all these genera to halichondrids. However, their precise placement is not possible based solely on morphological characters. Only a few previous molecular studies had included some species of this family. In the dataset of Morrow et al. (2012) [49] two partitions of the 28S rDNA gene highly supported the grouping of *Desmanthus* within Dictyonellidae *sensu* Morrow et al. (2012) [49]. This result was also supported by Redmond et al. (2013) [39] based on the analysis of the 18S rDNA. Here, we add for the first time an unlinked molecular marker, from the mtDNA CO1 gene, and support the assignment of *Desmanthus incrustans* to Dictyonellidae, and further, provide moderate support of a sister group relationship to the species *Dictyonella* sp. Based on morphological character analysis, Van Soest & Hajdu (2000) [80] suggested resurrecting the family Desmanthidae Topsent, 1893 within the ‘Lithistida’ demosponges for the genera *Desmanthus* and *Lithobubaris* ( = *Sulcastrella*) by excluding *Petromica.* Redmond et al. (2013) [39] already formally reallocated the genera *Desmanthus*, *Sulcastrella* and *Paradesmanthus* to Bubaridae. In contrast, our molecular data, based on the mtDNA CO1 gene, strongly recommend the reallocation of *Desmanthus* to Dictyonellidae, as proposed by Cárdenas et al. (2012) [6]. Since no molecular data for any species of the genera *Sulcastrella* and *Paradesmanthus* exists yet, for the time being we support their reallocation to the Bubaridae, as proposed by Redmond et al. (2013) [39]. Molecular data based on the 18S rDNA gene of the genus *Petromica* showed a close relationship to Halichondriidae *sensu* Morrow et al. (2012) [49]. Our analysis of mitochondrial CO1 sequences is consistent with their hypothesis. Additionally, our results display a strongly supported clade of the genus *Petromica* together with *Topsentia ophiraphidites* (Halichondriidae). This confirms earlier morphological findings of Van Soest & Zea (1986) [81]. Muricy et al. (2001) [82] amended the monophyly of *Petromica*, which is acknowledged in our molecular results, and showed support for the affinity with the Halichondriidae *sensu* Morrow et al. (2012). We therefore formally recommend reallocating *Petromica* close to halichondriids.

### Molecular phylogeny of desma-bearing astrophorids

#### Family Corallistidae

Our molecular results (28S rDNA, C1–D2 partition) concerning the relationships within ‘lithistids’ provide strong evidence that the monophyletic family Corallistidae is closely related to *Pleroma* of the family Pleromidae. This outcome was expected from morphological observations, due to the similarity of desma structures. Megaclone desmas of *Pleroma* and dicranoclone desmas of Corallistidae might have originated in the same way and only final stages differ in these desmas. Additionally, dichotriaenes occur as ectosomal spicules in both families. Interestingly, no other astrophorids group with this clade, affirming the persistent occurrence of dicranoclone and megaclone desmas since the Paleozoic. Our molecular data further indicate that *Herengeria auriculata* is the sister-taxon to *Isabella mirabilis.* This relationship is morphologically supported with the main differences being the possession of euaster-like microscleres in *Isabella mirabilis* from the Norfolk Ridge [51]. Additionally, we confirmed the non-monophyly (CO1 gene tree) of the genus *Isabella* as also shown in the recent study of Carvalho et al. 2014 [83]. The sister group relationship of the species *Herengeria vasiformis* to a clade containing *H. auriculata, Isabella mirabilis, Neoschrammeniella castrum* and *N. norfolki* is highly supported. The polyphyly of the genus *Herengeria* could be explained by evidence of differing gross morphologies between the two species, indicative of taxonomic divergence. *Herengeria vasiformis* is vase-shaped and *H. auriculata* is much more massive; and *H. vasiformis* has thicker microxeas and more massive and less regularly developed rhabd-like spirasters, as well as smaller spirasters, as described by Schlacher-Hoenlinger et al. (2005) [51]. *Neoschrammeniella norfolki* differs from *N. castrum* and other genera of the family Corallistidae by the presence of plesiasters and absence of microxeas. When the genus *Corallistes* was included in the analyses, monophyly of the family Corallistidae was not supported in the study of Redmond et al. (2013) [39]. However, a “*Corallistes* sp. (AY737636)” formed a clade with *Neophrissospongia microstylifera*, while a “*Corallistes* sp. (AJ224646)” was not found to be related to Theonellidae. This is likely a consequence of misidentification of this taxon and/or an inexact alignment compared to other sequences of Corallistidae. Considering all these aspects, the family Corallistidae should also be reallocated to Astrophorida.

#### Family Pleromidae

The family Pleromidae was recovered as polyphyletic, with *Pleroma menoui* closely related to Corallistidae and *Anaderma rancureli* to *Characella pachastrelloides* (Pachastrellidae). This is in agreement with our morphological character analysis, which also indicated its likely polyphyly. *Pleroma* lacks anatriaenes in contrast to *Anaderma*, which unequivocally includes them. Even though the relationship between *Anaderma* and *Characella* is not supported in our 28S rDNA gene tree, it might be conceivable based on the presence of similar morphological characters (e.g. anatriaenes) [68].

#### Family Macandrewiidae

The status of *Macandrewia* (Macandrewiidae) has been revised many times in the past, changing from affinities to Corallistidae [84] to belonging to *Callipelta* [32]. The possession of phyllotriaenes and desmas with triaenose crepis, however, supports a close relationship to other astrophorids. Due to the low variation within the 28S rDNA gene, it was not possible to determine the exact relationships with other lithistids or to astrophorid clades. Therefore, escalated taxon sampling, as well as gene sampling, needs to be improved in future to clarify the phylogenetic position of the family Macandrewiidae.

#### Family Phymaraphiniidae

The family Phymaraphiniidae contains three genera: *Exsuperantia* Özdikmen, 2009 [85], *Kaliapsis* Bowerbank, 1869 [86] and *Lepidothenea* de Laubenfels, 1936. Burton (1929) suggested a close relationship of *Exsuperantia* to Stellettidae due to its possession of phyllotriaenes. The original placement of *Exsuperantia* was with Theonellidae, due to similar ectosomal phyllotriaenes and microscleres as found in the genus *Racodiscula* (Theonellidae). However, the sculpture of the trider-like desmas (Fig. 3 G–H) clearly differentiate those two genera and families [87]. The only previous molecular analyses of *Exsuperantia* sp. did not support its close relationship with the tetraclone-bearing family Theonellidae [36]. Our results group *Exsuperantia* sp. as a sister to the astrophorid families Ancorinidae and Pachastrellidae, and the lithistid species *Anaderma rancureli*. However, neither BI nor ML values support this suggestion and so for the moment we allocate *Exsuperantia* to Astrophorida until further data is available. The phylogenetic position of the other two genera *Kaliapsis* and *Lepidothenea* will be the matter of further investigations.

#### Family Theonellidae

The family Theonellidae contains five genera: *Discodermia* du Bocage, 1869, *Manihinea* Pulitzer-Finali, 1993, *Racodiscula* Zittel, 1878, *Siliquariaspongia* Hoshino, 1981 and *Theonella* Gray, 1868. Theonellidae is characterized by ectosomal spicules ranging from phyllotriaenes to discotriaenes, choanosomal tetraclone desmas and microscleres as acanthorhabds, microxeas, streptasters and amphiasters. Due to the possession of triaenes Theonellidae was usually considered to group with astrophorid sponges [24], [25], [27]. More recently there has been increased interest in bioactive compounds from theonellids [18], with the genera *Discodermia* and *Theonella* receiving special attention and resulting in the amplification of four different gene regions for *Discodermia* and three for *Theonella* (see Tab.1). Previous phylogenetic reconstructions based on mtDNA CO1 and 28S rDNA have shown that Theonellidae is monophyletic [36]. This result was in contrast to those observed from the 18S rDNA analysis [39]. Our present molecular analyses of both gene regions (mtDNA CO1 and 28S rDNA) strongly support the monophyly of Theonellidae, and additionally the sister group relationship of *Theonella* to *Discodermia,* supporting the conclusions of Cárdenas et al. (2011) [36]. A sister group relationship of Theonellidae and Corallistidae as proposed by earlier morphological [27] and molecular analyses [14], is not supported by any of our gene trees.

#### Family Phymatellidae

The family Phymatellidae contains three valid extant genera: *Neoaulaxinia* Pisera & Lévi, 2002, *Neosiphonia* Sollas, 1888 and *Reidispongia* Lévi & Lévi, 1988. Tetraclone desmas and dichotriaenes are the characteristic megascleres for the family, while the three genera are differentiated by the possession of different microscleres. Until the present study no molecular data existed for this group, and so its precise placement among the astrophorids remained uncertain. Here we show for the first time the monophyly of the family and its genera, and suggest a close relationship with the astrophorid family Pachastrellidae. Similar triaenes found in both families would support this moderately supported molecular sister group relationship. We therefore propose reallocating the family Phymatellidae to the Astrophorida.

### Evolution of megascleres and microscleres in lithistid sponges

Our results suggest that desmas have evolved several times independently in different lithistid demosponge groups within the order Astrophorida. Furthermore, and conversely, secondary loss of desmas may have also occurred several times independently. However, the silica concentration of seawater has been shown to influence the development of spicules in demosponges [41], providing the possibility that if the silica concentration in seawater is low, desmas dis-articulate. So, if megascleres lose their function (e.g. as structural support for the cortex or as defense against predators), a secondary loss of megascleres is feasible. Microscleres have been lost frequently in the past within Tetractinellidae [36], [69].

## Conclusions

This study represents the first comprehensive molecular phylogenetic analysis of lithistid demosponges. We used two independent markers showing that at least 8 out of 13 lithistid families belong to the order Astrophorida. Further, we discovered Scleritodermidae and Siphonidiidae as a separate monophyletic group within the Tetractinellidae (Spirophorida+Astrophorida), however further investigation and inclusion of other spirophorids like Samidae and Spirasigmidae (not sampled here) is still pending in order to fully resolve the phylogenetic position of rhizoclone-bearing lithistids. We formally propose to reallocate most of the lithistid astrophorids. In addition, it is evident that Desmanthidae is polyphyletic and should be reallocated to their closest relatives within Halichondriidae. We also confirmed the sister-group relationship of the family Vetulinidae to Spongillida, and propose the resurrection of Sphaerocladina at the ordinal level to include both Recent and fossil taxa with obvious morphological apomorphies. Our suggested ancestral state reconstructions show possible secondary losses in spicule evolution within the desma-bearing astrophorids, and also indicate the possible deceptiveness of alleged morphological evidence for phylogenetic affinities based on non homologous characters, viz. flaws in the definition of particular spicule types (e.g. within the concept of “streptasters”), used historically as an important feature for sponge classification (see also Chombard et al. 1998 [42], [68] or Cárdenas & Rapp 2013 [69].

## Supporting Information

S1 Table **Morphological character matrix.**
(XLSX)

S1 File **Description of morphological data matrix (** **S1 Table** **).**
(DOCX)
