# Supplementary material for: Deceptive Desmas: Molecular Phylogenetics Suggests a New Classification and Uncovers Convergent Evolution of Lithistid Demosponges
Source: PLoS One. 2015 Jan 7;10(1):e116038. doi: 10.1371/journal.pone.0116038 (PMC4286238; doi:10.1371/journal.pone.0116038)
Supplement: S1 File — Description of morphological data matrix ( S1 Table ). (DOCX) [file pone.0116038.s002.docx]

**File S1: Description of morphological data matrix (Table S1)**

Morphological matrix of the Astrophorida/Lithistida species sampled in this study. Spirophorida outgroups are shaded in gray at the end of the Table. Present or absent Characters: **(1)** Sterrasters globular; **(2)** sterrasters flattened (=aspidasters); **(3)** amphiaster; **(4)** two types of amphiaster; **(5)** spiraster; **(6)** rhabd-like spiraster; **(7)** two types of spiraster; **(8)** streptaster; **(9)** large streptaster; **(10)** spinose microxeas; **(11)** microrhabds/microstrongyles; **(12)** microstyles; **(13)** acanthorhabds; **(14)** acanthoxeas; **(15)** thick microxeas in ectosome; **(16)** trichodragmas; **(17)** sanidasters; **(18)** microxeas in choanosom **(19)** sigmaspires; **(20)** anatriaene; **(21)** long shafted triaene; **(22)** short shafted triaene; **(23)** calthrops; **(24)** calthrop with reduced actine; **(25)** phyllotriaene; **(26)** discotriaene; **(27)** mesotriaene; **(28)** dichotriaene; **(29)** tetraclone; **(30)** dicranoclone; **(31)** megaclone; **(32)** trider; **(33)** triaenose crepis/monocrepidial; **(34)** euaster; **(35)** plesiaster.
